# A Novel Pentapeptide Inhibitor Reduces Amyloid Deposit Formation by Direct Interaction with hIAPP

**DOI:** 10.1155/2019/9062032

**Published:** 2019-01-29

**Authors:** Yue Shi, Wu Lv, Ao Jiao, Chengshuo Zhang, Jialin Zhang

**Affiliations:** ^1^Hepatobiliary Surgery Department and Unit of Organ Transplantation, The First Hospital of China Medical University, Shenyang 110001, China; ^2^Department of Geriatric Surgery, The First Hospital of China Medical University, Shenyang 110001, China; ^3^Department of General Surgery (VIP Ward), Liaoning Cancer Hospital & Institute, Cancer Hospital of China Medical University, Shenyang 110001, China

## Abstract

**Backgrounds:**

The presence of amyloid deposits of human islet amyloid polypeptide (hIAPP) in islet *β*-cells has been associated with type 2 diabetes occurrence and islet graft failure. Self-assembly into oligomers and fibrils during the process of aggregation by hIAPP can lead to failure and depletion of *β*-cells. Studies have shown that some critical regions of hIAPP might contribute to the aggregation. Thus, many studies focused on finding the effective molecules, especially the short-peptide inhibitors, that bind to these regions and disrupt the aggregation of hIAPP. In the present study, a novel pentapeptide inhibitor Phe-Leu-Pro-Asn-Phe (FLPNF) was designed and its effectiveness on the inhibition of the formation of amyloid deposits was examined.

**Methods:**

The binding mode between FLPNF and hIAPP was performed using molecular docking. The effectiveness of FLPNF on inhibiting hIAPP amyloid aggregation was tested by Thioflavin T (ThT) staining. Furthermore, negative stain electron microscopy was used to observe hIAPP fibrils. A biolayer interferometry analysis was used to identify the interaction between FLPNF and hIAPP. In addition, the cytotoxicity toward INS-1 cells was tested by a cell proliferation assay.

**Results:**

FLPNF was predicted to have a compact conformation to bind at the site of hIAPP. FLPNF strongly inhibited the amyloid aggregation of hIAPP at a 10 : 1 molar ratio *in vitro*. Coincubation of FLPNF with hIAPP decreased the amount of hIAPP fibrils. Furthermore, a direct interaction between FLPNF and hIAPP was confirmed. FLPNF could also decrease the cytotoxic effect of hIAPP.

**Conclusions:**

The novel pentapeptide inhibitor FLPNF was constructed and inhibited the aggregation through direct binding to hIAPP. It is considered a suitable inhibitor for hIAPP amyloid deposit formation.

## 1. Introduction

Human islet amyloid polypeptide (hIAPP), also known as amylin, is a kind of hormone secreted by *β*-cells consisting of 37 amino acid residues [1, 2]. It is cosecreted with insulin according to a fixed proportion in *β*-cells, which is stored in dense core secretory granules [3–5]. In physiological conditions, hIAPP regulates blood sugar levels by inhibiting the secretion of glucagon [6] and also has effects on delayed gastric emptying [7, 8]. Monomeric hIAPP is noncytotoxic, but the oligomers and fibrils formed during the aggregation process can cause failure and depletion of *β*-cells [9–12]. Lack of *β*-cells induces extremely low hIAPP secretion in type 1 diabetes, which in turn is not enough to form the aggregates [13, 14]. On the contrary, amyloid deposits of hIAPP contributed to the development of type 2 diabetes [15] and islet transplant failure in type 1 diabetes [13, 16].

Since hIAPP self-assembles to form amyloid deposits containing parallel *β*-sheet structure [17], it has been found that some amyloidogenic regions of hIAPP promote the formation of *β*-sheet aggregations. Studies have shown that sequence 8-20 or 30-37 of hIAPP is capable of forming fibrils itself [18], and so these are considered to be critical for the aggregation of hIAPP. In addition, sequence 20-29 is also a potential contributor for the formation of amyloid fibrils [19, 20]. Studies have shown that the *π*-*π* stacking effect of aromatic residues plays a key role in causing aggregation of hIAPP [21, 22]. In the early studies, short peptides were designed based on specific amino acid sequences of the amyloid-beta peptide (A*β*) in Alzheimer's disease. These could inhibit the *β*-sheet aggregations of A*β* and hence were named as beta-sheet breaker peptides [23, 24]. This evidence demonstrated that designing short-peptide inhibitors in accordance with the hIAPP's critical regions helps in inhibiting hIAPP aggregation.

So far, only few studies have examined short-peptide inhibitors for the inhibition of hIAPP. Peptides such as SNNFGA, GAILSS, NYGAILSS, and NFGAILPP can inhibit hIAPP aggregation *in vitro* [25, 26], while D-ANFLVH can reduce the formation of amyloid aggregates *in vivo* [27]. In the present study, a pentapeptide inhibitor Phe-Leu-Pro-Asn-Phe (FLPNF) that contains only five amino acids has been designed. It was designed based on 11-15 residues (RLANF) of hIAPP, because it was included in the critical amyloidogenic region 8-20 and has one aromatic amino acid phenylalanine (F). After that, the hydrophilic amino acid arginine (R) was replaced with another phenylalanine (F) to enhance the binding ability to hIAPP. Alanine (A) was substituted with a hydrophobic amino acid proline (P) to maintain good hydrophobicity. On the other hand, proline could suppress the pentapeptide forming *β*-strand to prevent self-assembly [28, 29]. Meanwhile, NFGAIL (residues 22–27 of hIAPP) was a peptide that could enhance hIAPP fibril formation [25] and was set as negative control. Thioflavin T (ThT) is a fluorochrome that binds to the folding of *β*-sheet protein deposits and changes the optical properties [30], which then shows a bright green fluorescence. In this paper, ThT staining was used to determine if FLPNF could inhibit hIAPP amyloid deposit formation in phosphate-buffered saline (PBS), thus protecting the INS-1 cells from hIAPP cytotoxicity. The morphological changes of hIAPP deposits were also observed by transmission electron microscopy (TEM).

## 2. Materials and Methods

### 2.1. Peptide Supply

Peptides hIAPP, FLPNF, and NFGAIL were synthesized by ChinaPeptide Co. Ltd. (Shanghai, China, purity > 98%). The synthetic peptides were solubilized in DMSO to make stock solutions with a final concentration of 10 mM hIAPP, 100 mM FLPNF, and 100 mM NFGAIL. The stock solutions were aliquoted and stored at -80°C according to the instructions.

### 2.2. Molecular Docking

A molecular docking study was performed to investigate the binding mode between FLPNF and hIAPP using Autodock Vina 1.1.2 [31]. The three-dimensional (3D) structure of hIAPP (PDB ID: 2L86) was downloaded from the RCSB Protein Data Bank (http://www.rcsb.org/). The 3D structure of FLPNF was built by the PyMOL 1.7.6 package (http://www.pymol.org/). The AutoDockTools 1.5.6 package [32, 33] was employed to generate the docking input files. FLPNF was prepared for docking by merging nonpolar hydrogen atoms and defining rotatable bonds. The search grid of the hIAPP site was identified as center_x = 3.756, center_y = 1.514, and center_z = 0.013 with dimensions size_x = 37.5, size_y = 33, and size_z = 21.75. In order to increase the docking accuracy, the value of exhaustiveness was set to 20. For Vina docking, the default parameters were used if it was not mentioned. The best scoring pose as judged by the Vina docking score was chosen and visually analyzed using the PyMOL 1.7.6 software.

### 2.3. Thioflavin T Fluorescence Assay

ThT (Sigma-Aldrich, Darmstadt, Germany) was used to stain the amyloid deposits of hIAPP in 96-well plates. A volume of 0.1 *μ*L of each stock solution of hIAPP and FLPNF was added to 100 *μ*L of PBS (pH = 7.4, containing 20 *μ*M ThT), and this group was set as “hIAPP+FLPNF.” Similarly, 0.1 *μ*L stock solution of hIAPP and NFGAIL added to 100 *μ*L of PBS (containing 20 *μ*M ThT) was set as “hIAPP+NFGAIL.” Meanwhile, 0.1 *μ*L stock solution of hIAPP with additional 0.1 *μ*L DMSO was added to 100 *μ*L PBS (containing 20 *μ*M ThT), and this was set as “hIAPP.” The PBS solution with 20 *μ*M ThT and 0.2 *μ*L DMSO was set as the control. The plates were incubated at 37°C for 0, 12, 24, and 48 hours. Finally, the fluorescence image was detected by an XDY-100 fluorescence microscope (Zhongheng, Shanghai, China). Subsequently, the fluorescence intensity values of each group were measured by a Fluoroskan Ascent FL luminescence meter (Thermo Scientific, USA) using an excitation *λ* of 435 nm and an emission *λ* of 485 nm. At each time course, the fluorescence intensity values of the control group were set as 1, while the values of test groups relative to the control group were set as fluorescence intensity (A.U.) and used for statistical analysis. Each experiment was repeated thrice.

Different concentrations (0, 20, 50, 100, 200, and 400 *μ*M) of FLPNF or NFGAIL were added into PBS with 10 *μ*M hIAPP at the same conditions mentioned above to verify their inhibitory effects. The fluorescence images and the fluorescence intensity values of each group were collected after incubating for 24 hours. Each experiment was repeated thrice.

### 2.4. Transmission Electron Microscopy

TEM was used to directly observe the relative density and morphological changes of hIAPP amyloid fibrils. PBS solution containing 10 *μ*M hIAPP alone (hIAPP), in combination with 100 *μ*M FLPNF (hIAPP+FLPNF), or in combination with 100 *μ*M NFGAIL (hIAPP+NFGAIL) was incubated for 24 hours at 37°C. A volume of 10 *μ*L of each sample was placed on mesh copper grids (Servicebio, Wuhan, China) and was covered by a carbon-stabilized formvar film. After 3 min, excess fluid was removed, and the grids were stained with 2% phosphotungstic acid for 3 min. Samples were analyzed using a HT7700 transmission electron microscope (Hitachi, Japan).

### 2.5. Biolayer Interferometry

Interaction between FLPNF and hIAPP was studied using Octet K2 biolayer interferometry (Pall ForteBio, USA). Experiments were performed by Shuangtian Biotech Co. Ltd. FLPNF were reconstituted in PBS (pH = 7.4) and immobilized on aminopropylsilane (APS) biosensors. Following 30 sec equilibration of immobilized FLPNF in PBS, the interaction with the target protein hIAPP (400 *μ*M) was performed for 300 sec. This was followed by a 600 sec dissociation phase in dissociation buffer (50% DMSO+0.05% PBST) in PBS. A response unit represented the bonding height on the biosensor. The association and dissociation curves were analyzed and constructed by Octet K2 biolayer interferometry.

### 2.6. Cell Proliferation Assay (MTS)

INS-1 cells were purchased from BioLeaf Biotech Co. Ltd. (Shanghai, China). INS-1 cells were maintained as described previously [34]. The INS-1 cells at a density of 2 × 10^4^ were plated in a 96-well plate. Twenty-four hours later, the stock solutions of hIAPP, FLPNF, and NFGAIL were diluted by fresh medium (100 *μ*L fresh medium in each well). The cells were incubated with fresh medium containing 10 *μ*M hIAPP alone, 10 *μ*M hIAPP with 100 *μ*M FLPNF, or 10 *μ*M hIAPP with 100 *μ*M NFGAIL for additional 24 hours. The untreated control cells received equal fresh medium. The medium of each group contained 0.2 *μ*L DMSO, equal to 0.2% DMSO. Consequently, 10 *μ*L MTS solution was added to each well and incubated for additional 2 hours. The optical density (OD) values were measured at 490 nm using a PowerWave XS2 microplate spectrophotometer (Thermo Scientific, USA). Each experiment was repeated thrice.

### 2.7. Statistical Analysis

GraphPad Prism 7.0 software (San Diego, CA, USA) was used for statistical analysis. Data are presented as means ± standard deviations (SD). Student's *t*-test was performed for statistical analysis. *P* value < 0.05 was considered to be statistically significant.

## 3. Results

### 3.1. Molecular Docking Results

**Figure 1:**
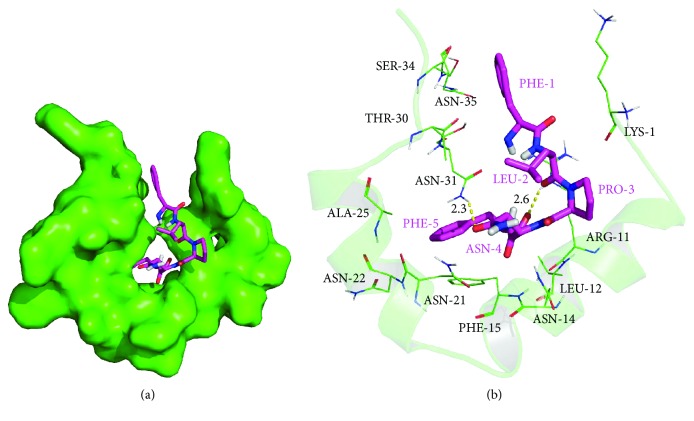
Molecular docking simulation of the interactions between FLPNF and hIAPP. (a) FLPNF was docked into the binding site of hIAPP (total view). (b) Detailed view of the binding mode between FLPNF and hIAPP. hIAPP was represented with cartoon, and the representative binding residues were shown in lines; FLPNF was represented with rose red sticks. The hydrogen bonds were shown as yellow dotted lines.

The peptide FLPNF was docked into the binding site of hIAPP, and the results are shown in Figure 1. The maximum binding affinity between FLPNF and hIAPP was predicted to be -6.4 kcal/mol. FLPNF adopted a compact conformation to bind at the site of hIAPP (Figure 1(a)). The residue Phe-5 of FLPNF was located at the hydrophobic site, surrounded by the residues Leu-12, Phe-15, and Ala-25 of hIAPP, forming stable hydrophobic bindings (Figure 1(b)). Detailed analysis showed that the residue Phe-1 of FLPNF formed cation-*π* interactions with the residues Lys-1 and Arg-11 of hIAPP, while the side chain of the residue Phe-5 of FLPNF formed a *π*-*π* stacking interaction with the residue Phe-15 of hIAPP. Importantly, two hydrogen bond interactions were observed between the residues Asn-4 and Phe-5 of FLPNF and the residues Asn-31 (bond length: 2.3 Å) and Arg-11 (bond length: 2.6 Å) of hIAPP, respectively, which were the main interaction between them (Figure 1(b)). All these predicted interactions might help FLPNF to anchor in the binding site of hIAPP.

### 3.2. Effects of FLPNF on Inhibiting hIAPP Amyloid Formation

**Figure 2:**
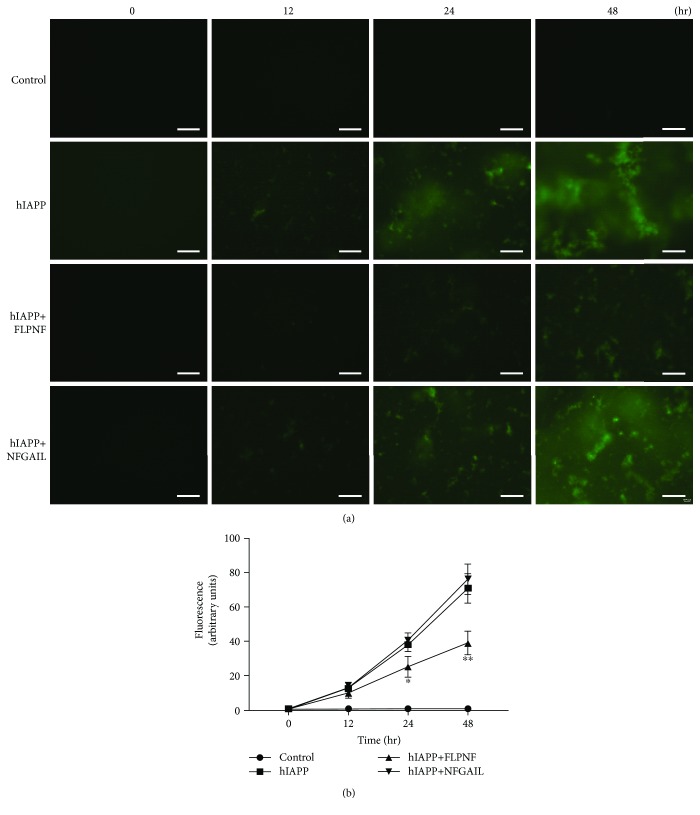
Quantitative analysis of the inhibitory effects of FLPNF and NFGAIL on hIAPP aggregation formation by Thioflavin T (ThT) staining. (a) ThT fluorescence of each group was observed at 0, 12, 24, and 48 hours using a fluorescence microscope (“control” represented PBS solution, “hIAPP” represented 10 *μ*M hIAPP in PBS, “hIAPP+FLPNF” represented 10 *μ*M hIAPP with 100 *μ*M FLPNF in PBS, and “hIAPP+NFGAIL” represented 10 *μ*M hIAPP with 100 *μ*M NFGAIL in PBS; 20 *μ*M ThT was added to each group; scale bar: 50 *μ*m). (b) ThT fluorescence intensity values were measured by a luminescence meter at 0, 12, 24, and 48 hours (values were presented as means ± SD; each experiment was repeated thrice; ^∗^significantly different compared to hIAPP, unpaired *t*-test, *P* < 0.05; ^∗∗^significantly different compared to hIAPP, unpaired *t*-test, *P* < 0.01).

The ability of the peptides FLPNF and NFGAIL to inhibit hIAPP aggregation in PBS was examined by the ThT fluorescence assay. After 12-hour incubation with hIAPP (10 *μ*M), a gradual increase in flocculence and strip-like green fluorescence was observed. Meanwhile, the fluorescence signal was similar to that of the hIAPP group after adding NFGAIL (100 *μ*M) at each time point. In addition, a strong reduction of the fluorescence signal was observed at 24 and 48 hours after the addition of FLPNF (100 *μ*M). There was no green fluorescence observed in the control group at any time (Figure 2(a)).

Afterwards, the fluorescence intensity was determined using a luminescence meter. No fluorescence signal was detected in the control group over time. The addition of FLPNF (100 *μ*M), followed by incubation with hIAPP, decreased the fluorescence intensity compared to hIAPP alone at 12, 24, and 48 hours. The fluorescence signal was significantly lowered in the hIAPP+FLPNF group compared to the hIAPP group at 24 h (*P* < 0.05) and 48 h (*P* < 0.01). The fluorescence intensity did not decrease in the hIAPP+NFGAIL group compared with the hIAPP group (Figure 2(b)).

**Figure 3:**
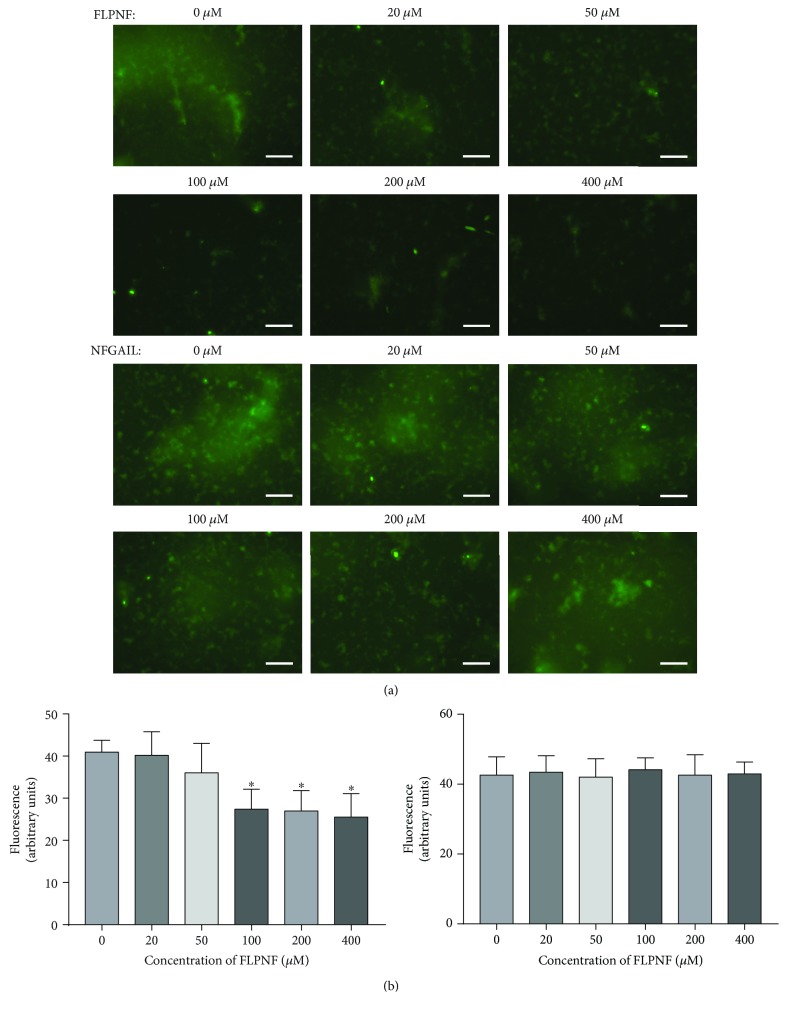
Analysis of inhibitory effects of different concentrations of FLPNF and NFGAIL on hIAPP aggregation formation by ThT staining. 10 *μ*M hIAPP incubated with 0, 20, 50, 100, 200, and 400 *μ*M FLPNF or NFGAIL in PBS for 24 hours at 37°C. (a) ThT fluorescence of each group was observed using a fluorescence microscope (stained by 20 *μ*M ThT; scale bar: 50 *μ*m). (b) The fluorescence intensity values of each group were measured by a luminescence meter for 24 hours (values were presented as means ± SD; each experiment was repeated thrice; ^∗^significantly different compared to 0 *μ*M, unpaired *t*-test, *P* < 0.05).

Since FLPNF had inhibitory effects at tenfold molar excess of hIAPP, the relationship between the inhibitory effects and the concentration of FLPNF was further verified using ThT staining. With the increasing concentrations (0, 20, 50, 100, 200, and 400 *μ*M) of FLPNF, followed by incubation with 10 *μ*M hIAPP for 24 hours at 37°C, the green fluorescence signal as observed by a fluorescence microscope was gradually decreased (Figure 3(a)). Meanwhile, the fluorescence intensity values were gradually reduced; in particular, the fluorescence intensity values in the 100, 200, and 400 *μ*M groups were significantly lowered compared to that in the 0 *μ*M group (*P* < 0.05) (Figure 3(b)). On the other hand, the fluorescence signal showed no reduction after adding any concentration of NFGAIL compared to the 0 *μ*M group (Figures 3(a) and 3(b)).

### 3.3. Observation of Reduction of hIAPP Amyloid Fibril Formation by FLPNF

**Figure 4:**
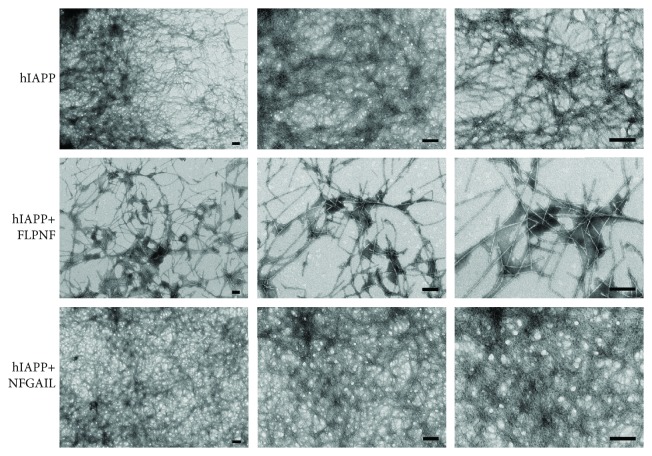
Observation of morphology of hIAPP fibril formation by a transmission electron microscope (TEM). The electron micrographs were taken following incubation for 24 hours at 37°C. The concentration of hIAPP was 10 *μ*M while the concentrations of FLPNF and NFGAIL were 100 *μ*M. Full-length hIAPP fibrils showing typical morphology and formation of dense aggregates. The fibrils of hIAPP incubated with FLPNF were not completely prevented but were reduced in density. No reduction of the fibrils was observed after adding NFGAIL. Scale bar: 200 nm.

To confirm the above results, we used a TEM to observe the effect of FLPNF on the inhibition of hIAPP amyloid fibril formation. The results showed that incubation with hIAPP (10 *μ*M) alone in PBS demonstrated typical dense fibrils by TEM after 24 hours, while the addition of FLPNF (100 *μ*M) reduced the amounts of fibril formation. On the contrary, the addition of NFGAIL (100 *μ*M) did not reduce or even increase the amount of the fibril formation observed by naked eyes (Figure 4). These results demonstrated that FLPNF had inhibitory effects on the formation of hIAPP aggregation *in vitro*.

### 3.4. FLPNF Could Directly Interact with hIAPP

**Figure 5:**
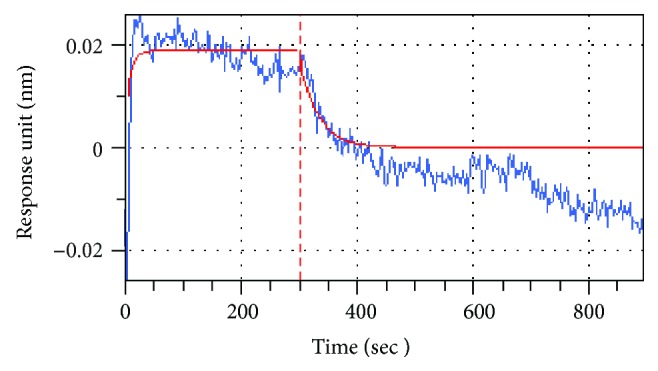
Association between FLPNF and hIAPP. Representative curve obtained using biolayer interferometry showing the level of interaction between immobilized FLPNF and hIAPP (100 *μ*M) in PBS. During the first 300 seconds, the association curve represented a rapid increase in response units. Over the next 600 seconds, the response units were slowly dropped to 0.

Next, biolayer interferometry was used to test whether FLPNF exerts inhibitory effects by direct binding to hIAPP. As shown in Figure 5, interaction of hIAPP with FLPNF immobilized on a biosensor showed a rapid increase in the response units (approximately 0.02 nm) and was kept at a certain level over 300 sec on the association curve. On the dissociation curve, the interaction was tardily reversible. Briefly, a strong interaction between FLPNF and hIAPP was found. These results indicated that FLPNF inhibited the hIAPP amyloid formation by a direct interaction with hIAPP.

### 3.5. FLPNF Inhibited the hIAPP Cytotoxic Effect in INS-1 Cells

**Figure 6:**
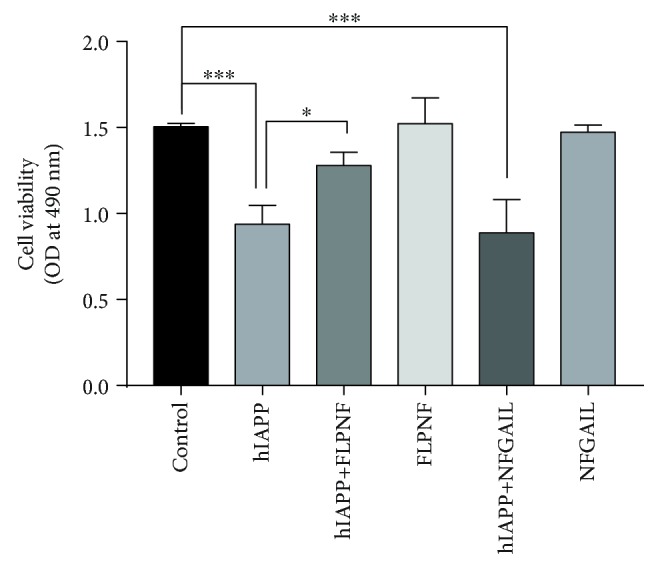
Inhibition of hIAPP cytotoxicity in INS-1 cells by adding FLPNF or NFGAIL. The cell viability was measured by the MTS assay. “Control” means INS-1 cells incubated in medium alone. The concentration of hIAPP added was 10 *μ*M, while the concentrations of FLPNF and NFGAIL were 100 *μ*M (the OD values read at 490 nm were presented as means ± SD; each experiment was repeated thrice; unpaired *t*-test, ^∗∗∗^*P* < 0.001; ^∗^*P* < 0.05).

Considering that the aggregation of hIAPP could lead to the failure of islet *β*-cells [35], we examined whether FLPNF could decrease the cytotoxic effects of hIAPP in INS-1 cells. The MTS assay was used to measure the viability of cells. As shown in Figure 6, the addition of hIAPP (10 *μ*M) to INS-1 cells significantly decreased the cell viability compared to the control group (*P* < 0.001). The presence of FLPNF (100 *μ*M) with hIAPP demonstrated a significant improvement in cell viability compared to the hIAPP group (*P* < 0.05). Moreover, FLPNF alone showed no direct effect on the viability of INS-1 cells. Adding NFGAIL (100 *μ*M) showed no effect of reducing the cytotoxic effects of hIAPP (Figure 6).

## 4. Discussion

hIAPP is the major component of amyloid deposition in the islets of type 2 diabetes [15] and contributes to the islet transplant failure in type 1 diabetes [13, 16]. The oligomers and fibrils formed by aggregation of hIAPP can cause loss of *β*-cells [9–12]. The conformation of hIAPP changes from a random coil to a *β*-sheet, thus promoting the formation of amyloid fibrils [36–38]. Therefore, compounds preventing hIAPP aggregation would enhance *β*-cell survival [39]. Short-peptide inhibitors interact with certain regions of amyloid proteins to block the conformational changes [23], which are considered the initial steps in hIAPP aggregation [38]. Accordingly, we focused on designing a short-peptide inhibitor to prevent the aggregation of hIAPP by blocking *β*-sheet formation. In this study, the molecular simulations gave us the most likely interaction mode and the binding site between FLPNF and hIAPP. With tenfold molar excess to hIAPP, FLPNF partially inhibited amyloid formation in PBS solution for 24 hours. This result was also confirmed by observing the formation of hIAPP fibrils using TEM. Biolayer interferometry verified the direct interaction of FLPNF with hIAPP. In addition, FLPNF decreased the cytotoxic effect of hIAPP. Our results have demonstrated that the pentapeptide FLPNF could be an effective agent for the treatment of hIAPP aggregation *in vitro*.

There were only few studies that investigated the effective peptide inhibitors for the treatment of hIAPP aggregation *in vitro* including SNNFGA, GAILSS, NYGAILSS, and NFGAILPP [25, 26]. But there were few limitations: SNNFGA had poor hydrophobicity, GAILSS was unstable as predicted by the software (ProtParam tool, https://web.expasy.org/protparam/), and the molecular weights of NYGAILSS and NFGAILPP were large (823.9 and 827.9, respectively). D-ANFLVH could reduce the islet amyloid accumulation *in vivo* with good characteristics [27]. Even so, FLPNF was optimally designed and consisted of only five amino acids. Its molecular weight (636.75) was less than that of D-ANFLVH (699.81), which remained easy to synthesize. FLPNF was also in good hydrophobicity and stable as predicted.

FLPNF showed an inhibitory effect on hIAPP amyloid deposit formation by ThT staining. Also, TEM was used to observe the hIAPP fibrils to confirm the effect of FLPNF, where the fibrils were less numerous but appeared to be more thickened when FLPNF was added. This suggested that the interaction with hIAPP altered normal fibril assembly and warranted further investigation. The obtained results showed that FLPNF could reduce hIAPP aggregation but still could not completely prevent the formation of fibrils. In the early research, SNNFGA and GAILSS could not completely prevent the formation of fibrils of hIAPP on TEM images [25]. So, it showed that the peptide inhibitors could maintain hIAPP in a monomeric form for a longer period, thereby attenuating the formation of fibrils. Further research is needed to improve the inhibitory effect of FLPNF.

Since there was no other factor involved in the PBS environment, we speculated that the mechanism of FLPNF inhibiting hIAPP aggregation occurred through binding to key regions of hIAPP. The molecular docking simulations gave us rational explanation of the interactions between FLPNF and hIAPP, which provided valuable information for the study of the mechanism of action between them at the molecular level. Since the *π*-*π* stacking effect of aromatic residues plays a key role in aggregation of hIAPP [21, 22], the predicted cation-*π* interactions and the *π*-*π* stacking interaction between FLPNF and hIAPP might disrupt the self-assembly of hIAPP and were consistent with our original principles of designing a short-peptide inhibitor. Subsequently, biolayer interferometry was used to confirm that there was a direct interaction between them. Previous studies have not performed experiments to verify the mechanism of short-peptide inhibitors, but we provided experimental evidence and produced an ideal treatment strategy for hIAPP aggregation. Further research is still needed to confirm the specific interaction mode between FLPNF and hIAPP. Moreover, the results also showed that the residues 11-15 of hIAPP had a remarkable effect on fibrillogenesis.

FLPNF decreased the cytotoxic effect of hIAPP and then improved the cell viability of rat insulinoma cells. Meanwhile, FLPNF alone had no direct effect on cell viability toward INS-1 cells. This result remained interesting as FLPNF is a small and nontoxic compound and can be used for the inhibition of hIAPP aggregation. Nonetheless, the cell viability was still lower when hIAPP was incubated with FLPNF at a 1 : 10 ratio compared to the control group, possibly suggesting that the small cytotoxic aggregates could still be formed in the presence of FLPNF. These findings can be important for the refinement of future inhibitory molecules. In addition, we only measured the viability of cells within 24 hours because INS-1 cells were rapidly dying after 24 hours of incubation with 10 *μ*M hIAPP and were almost completely dead after 48 hours. In future, we plan to construct the INS-1 cell line expressing hIAPP to further study the function of peptide inhibitors. Further investigation regarding the ways of FLPNF inhibition on hIAPP aggregation in cell lines is warranted. Peptides may be used *in vivo* by intraperitoneal [27] or intravenous [40] injection as reported. We will establish the hIAPP transgenic mice to examine the inhibitory effect of FLPNF *in vivo* in the future.

There are still some limitations about the pentapeptide; the half-life *in vitro* is predicted as 1.1 hours by the software (ProtParam tool, https://web.expasy.org/protparam/). However, our results showed that FLPNF still had an inhibitory effect at 48 hours *in vitro*. In the future, the half-life and the inhibitory effect of FLPNF *in vivo* need to be verified. Moreover, we also consider modifying this peptide to improve its stability, such as modification by PEGylation [41, 42]. This might be conducive to promoting the clinical application of FLPNF. Further *in vivo* investigations are needed.

Taken together, our results have demonstrated that the optimal designed pentapeptide FLPNF is capable of inhibiting hIAPP fibril formation *in vitro* and partially protecting INS-1 cells from the cytotoxic effect of hIAPP. Our research laid a foundation for future experiments in cell lines and in rat models. Nonetheless, the inhibitory effect needs to be further refined. The present study provided new insights into potential therapeutic compounds for the treatment of hIAPP aggregation.

## Data Availability

All data included in this study are available from the corresponding author upon request.
